# Endoscopic ultrasound-guided fine-needle aspiration for the diagnosis of peritoneal tuberculosis

**DOI:** 10.1055/a-2719-2960

**Published:** 2025-11-04

**Authors:** Yuping Zhang, Yan Zhang, Yifan Xu, Guihui Zhang, Haiyan Dong, Guang Zhang

**Affiliations:** 1 66310 Department of Gastroenterology, The First Affiliated Hospital of Shandong First Medical University and Shandong Provincial Qianfoshan Hospital, Jinan, China; 2 66310 Department of Radiology, The First Affiliated Hospital of Shandong First Medical University and Shandong Provincial Qianfoshan Hospital, Jinan, China; 3 66310 Department of Pathology, The First Affiliated Hospital of Shandong First Medical University and Shandong Provincial Qianfoshan Hospital, Jinan, China; 4 66310 Department of Health Management, The First Affiliated Hospital of Shandong First Medical University and Shandong Provincial Qianfoshan Hospital, Jinan, China; 5 Shandong Engineering Research Center of Health Management, Jinan, China

We report the case of a 27-year-old male patient admitted to the hospital with recurrent periumbilical pain for 4 months, accompanied by fever, cough, and diarrhea. Contrast-enhanced CT revealed a right upper lung lobe nodule with potential metastatic involvement, circumferential gastric wall thickening at the cardia and greater curvature suspicious for malignancy, multiple abnormal intraabdominal/peritoneal/omental densities indicative of metastatic dissemination, accompanied by ascites and pelvic effusion ( Fig. 1 ).

**Fig. 1:**
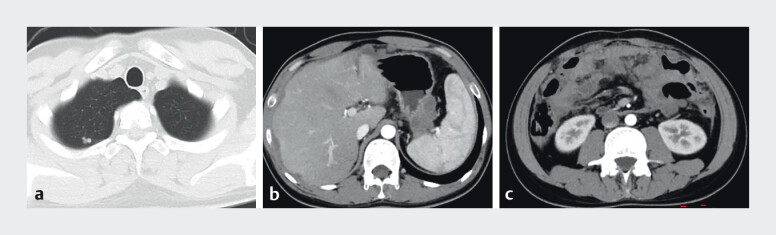
Contrast-enhanced CT revealed a right upper lung lobe nodule ( **a** ), circumferential gastric wall thickening at the cardia and greater curvature ( **b** ), multiple abnormal intraabdominal/peritoneal/omental densities, accompanied by ascites and pelvic effusion ( **c** ).

To further confirm the diagnosis, the patient underwent gastrointestinal endoscopy. Esophagogastroduodenoscopy revealed multiple gently sloping elevations (2–3 cm in diameter) in the gastric body and antrum, while colonoscopy identified a solitary 1-cm gently sloping elevation in the descending colon. All lesions demonstrated firm consistency with restricted mobility upon instrumental palpation, each showing intact overlying mucosa ( Fig. 2 ). Endoscopic ultrasonography (EUS) revealed multiple hypoechoic masses (1–5 cm) with moderate stiffness in the gastric/duodenal walls, splenic hilum, and perihepatic regions. These lesions demonstrated ill-defined margins, absence of detectable vascularity on Doppler imaging, and heterogeneous internal architecture containing flocculent hyperechoic foci and nodular hyperechoic deposits. Contrast-enhanced harmonic EUS showed hypoenhancement patterns ( Fig. 3 ). To obtain pathological specimens for definitive diagnosis, sequential endoscopic ultrasound-guided fine-needle aspiration (EUS-FNA) of perihepatic, gastric wall, and perisplenic hilar lesions was performed with a single pass per lesion, yielding viscous yellowish purulent strands suggestive of necrotic material ( Video 1 ).

**Fig. 2:**
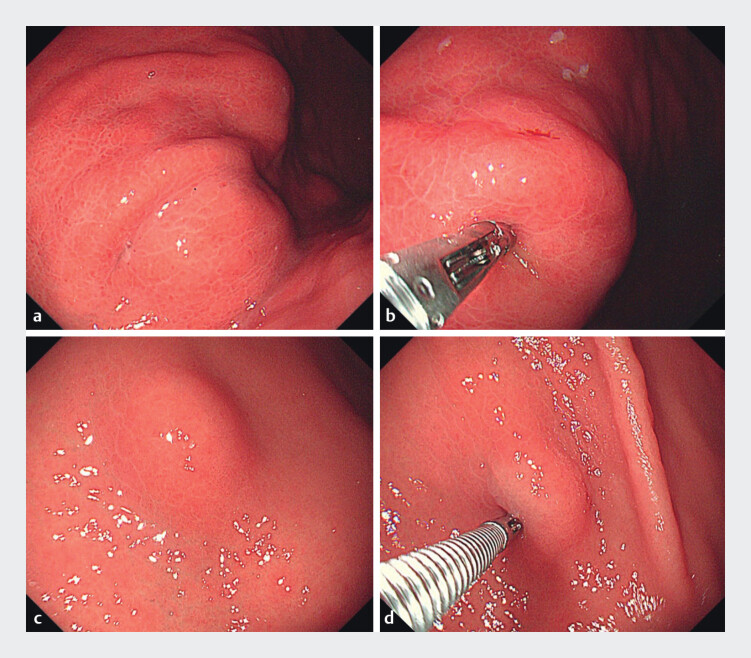
Esophagogastroduodenoscopy (EGD) revealed multiple gently sloping elevations (2–3 cm in diameter) in the gastric body and antrum with intact overlying mucosa ( **a** , **c** ). All lesions demonstrated firm consistency with restricted mobility upon instrumental palpation ( **b** , **d** ).

**Fig. 3:**
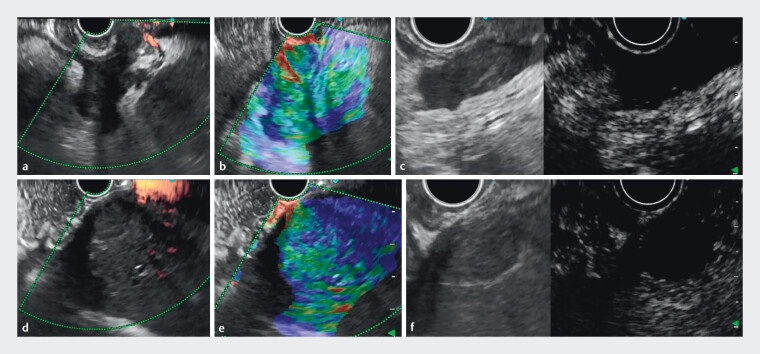
Endoscopic ultrasonography (EUS) revealed multiple hypoechoic masses (1–5 cm) with moderate stiffness in the gastric/duodenal walls, splenic hilum ( **a** – **c** ), and perihepatic regions ( **d** – **f** ). These lesions demonstrated ill-defined margins, absence of detectable vascularity on Doppler imaging ( **a** , **d** ), and heterogeneous internal architecture containing flocculent hyperechoic foci and nodular hyperechoic deposits ( **b** , **e** ). Contrast-enhanced harmonic EUS (CH-EUS) showed hypoenhancement patterns ( **c** , **f** ).

Definitive diagnosis was confirmed by endoscopic ultrasound-guided fine-needle aspiration (EUS-FNA) in a patient with peritoneal tuberculosis.Video 1

Histopathological analysis demonstrated necrotizing granulomas with caseous necrosis ( Fig. 4 ), positive for acid-fast bacilli on fluorescent staining (Ziehl–Neelsen method) and Mycobacterium tuberculosis complex DNA detection via quantitative real-time PCR (cycle threshold <25), confirming the diagnosis of tuberculosis.

**Fig. 4:**
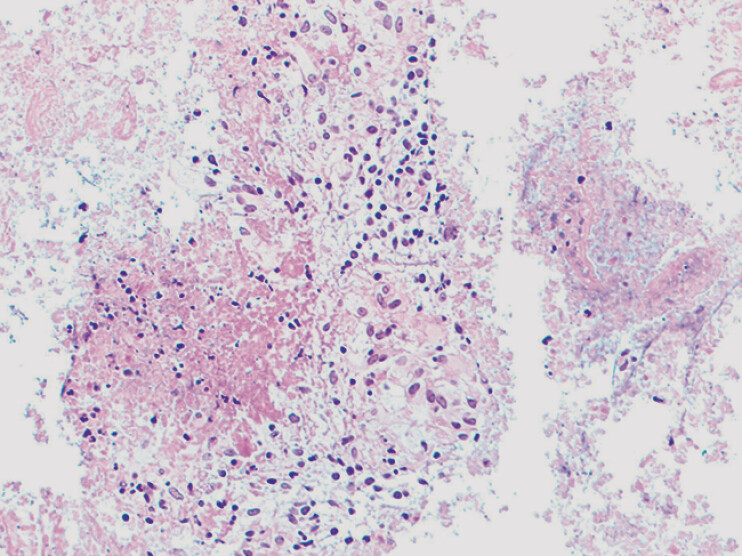
Histopathological analysis demonstrated necrotizing granulomas with caseous necrosis.

Peritoneal tuberculosis, an uncommon extrapulmonary manifestation of *Mycobacterium tuberculosis* infection, remains a significant diagnostic challenge due to persistent difficulties in clinical recognition and microbiological confirmation. EUS-FNA provides sufficient samples safely and efficiently for further cytology, histopathology, and microbial examinations.

Endoscopy_UCTN_Code_CCL_1AF_2AG_3AD

## Notes

Endoscopy E-Videos https://eref.thieme.de/e-videos
*E-Videos* is an open access online section of the journal *Endoscopy* , reporting on interesting cases and new techniques in gastroenterological endoscopy. All papers include a high-quality video and are published with a Creative Commons CC-BY license. Endoscopy E-Videos qualify for HINARI discounts and waivers and eligibility is automatically checked during the submission process. We grant 100% waivers to articles whose corresponding authors are based in Group A countries and 50% waivers to those who are based in Group B countries as classified by Research4Life (see: https://www.research4life.org/access/eligibility/ ). This section has its own submission website at https://mc.manuscriptcentral.com/e-videos .

